# Circadian clocks, feeding time, and metabolic homeostasis

**DOI:** 10.3389/fphar.2015.00112

**Published:** 2015-05-27

**Authors:** Georgios K. Paschos

**Affiliations:** Department of Systems Pharmacology and Translational Therapeutics, Institute for Translational Medicine and Therapeutics, University of PennsylvaniaPhiladelphia, PA, USA

**Keywords:** circadian clock, metabolic homeostasis, circadian misalignment, feeding time, circadian rhythms

## Abstract

Metabolic processes exhibit diurnal variation from cyanobacteria to humans. The circadian clock is thought to have evolved as a time keeping system for the cell to optimize the timing of metabolic events according to physiological needs and environmental conditions. Circadian rhythms temporally separate incompatible cellular processes and optimize cellular and organismal fitness. A modern 24 h lifestyle can run at odds with the circadian rhythm dictated by our molecular clocks and create desynchrony between internal and external timing. It has been suggested that this desynchrony compromises metabolic homeostasis and may promote the development of obesity (Morris et al., 2012). Here we review the evidence supporting the association between circadian misalignment and metabolic homeostasis and discuss the role of feeding time.

Life on earth has adapted to our world of days and nights by evolving molecular mechanisms anticipating the most advantageous time of day for biological processes. In mammals, these daily rhythms are maintained by autoregulatory transcriptional and translational feedback loops involving the basic helix loop helix PER-ARNT-SIM (bHLH/PAS) transcription factors BMAL1, CLOCK, and NPAS2. BMAL1 heterodimerizes with either CLOCK or NPAS2 and drive transcription through E-boxes located within the promoters of numerous target genes. Among the target genes are Period homolog (Per1-3), Cryptochrome (Cry1-2) and Rev-erbα that encode repressors of the BMAL1: CLOCK/NPAS2 transcriptional activity. After a delay, the translated PER and CRY proteins heterodimerize, translocate to the nucleus, and repress BMAL1: CLOCK/NPAS2 heterodimers. The PER and CRY heterodimers are progressively degraded, allowing the circuit to start again. This negative feedback leads to a cycle in gene expression that takes approximately 24 h to complete (Ukai and Ueda, 2010). Post-translational modifications of the proteins of the circuit generate the essential time delay that maintains the period of the cycle at approximately 24 h (Crane and Young, 2014). As a result, BMAL1: CLOCK/NPAS2 bind to DNA in a rhythmic manner leading to rhythmic expression of target genes (Koike et al., 2012). Additional feedback pathways by nuclear receptors retinoid-related orphan receptor alpha (RORα) (Sato et al., 2004), peroxisome proliferator–activated receptor gamma (PPARγ) (Yang et al., 2012) and peroxisome proliferator-activated receptor gamma coactivator 1-alpha (PGC-1α) (Liu et al., 2007) provide further robustness to the circuit. The circadian system is organized in a hierarchical manner with a master clock located at the suprachiasmatic nucleus (SCN) of the hypothalamus. The SCN receives photic input through direct retinal innervation that initiates gene expression in the SCN (Hastings and Herzog, 2004). In this way, light exposure entrains the SCN clock to solar time, adjusting the oscillator to a precise 24 h cycle (Khalsa et al., 2003). The master clock of the SCN communicates day-night information to the rest of the body. Through neuronal and humoral signals, the SCN sends this information to peripheral circadian clocks that exist in almost all cells of the rest of the body and synchronize them to the same phase (Mohawk et al., 2012). Whereas light is the dominant timing cue for the SCN oscillator, the clocks of the periphery respond to other environmental cues such as temperature (Glaser and Stanewsky, 2007) and food intake (Damiola et al., 2000) and alter their phase accordingly.

The notion that running at odds with the timing imposed by the master pacemaker (the term “circadian clock” will be used for the rest of the manuscript) results in inefficiency in energy expenditure and obesity has been supported by epidemiological studies. Circadian misalignment has been associated with an increased prevalence of obesity and diabetes. The prevalence of obesity is higher among night-shift workers compared to day workers, and chronic shift work is positively associated with body mass index (BMI) (Karlsson et al., 2001; Parkes, 2002; Di Lorenzo et al., 2003; Ostry et al., 2006; Pan et al., 2011). Prospective studies of healthy volunteers undergoing a 6-day simulated shiftwork protocol show a reduction of energy expenditure in response to the shiftwork (Mchill et al., 2014). Certain sleep disorders also generate misalignment between the rhythms imposed by the circadian clock and behavioral rhythms. Patients with sleep disorders have a higher risk for developing obesity (Phillips et al., 2000; Liu et al., 2013), and the duration of sleep is inversely correlated with body weight in healthy men and women (Patel et al., 2006, 2008; Cappuccio et al., 2008; Chen et al., 2008; Mozaffarian et al., 2011). Prospective study of sleep deprivation shows an increase in body weight after 5 days of insufficient sleep, characterized by an increase in food intake at night (Markwald et al., 2013). A 12-h shift of the sleep/wake and fasting/feeding cycle compared with the central circadian system, while maintaining an isocaloric diet, reduces glucose tolerance, increases blood pressure, and decreases the satiety hormone leptin (Scheer et al., 2009). Exposure of human volunteers to a 28 h day as a mean for circadian disruption in combination with sleep deprivation results in reduced resting metabolic rate and increased post-prandial glycemia as a result of reduced pancreatic insulin secretion (Buxton et al., 2012).

The metabolic impact of circadian misalignment has been studied in animals. The link between the circadian clock and metabolism first emerged from transcriptome analysis of mouse suprachiasmatic nuclei and liver (Panda et al., 2002). Panda et al. showed rhythmically expressed genes encoding regulators and enzymes from multiple metabolic pathways, especially cholesterol synthesis and gluconeogenesis, and suggested that the expression of these genes is under the control of the circadian clock (Panda et al., 2002). Since that study, amino acids and fatty acids were found to oscillate in both mouse liver (Eckel-Mahan et al., 2012) and human plasma (Dallmann et al., 2012). Studies in animal models of circadian clock disruption provide evidence for the requirement of circadian rhythms for metabolic fitness. Early studies showed that gluconeogenesis is impaired in Bmal1 knockout mice and Clock Δ19 mutants, resulting in loss of the circadian variation in the recovery of blood glucose in response to insulin (Rudic et al., 2004). Zhang et al. showed that Cry1 inhibits hepatic gluconeogenesis by blocking adenyl cyclase signaling in response to glucagon (Zhang et al., 2010). Hepatic overexpression of Cry1 improves sensitivity to insulin in db/db pro-diabetic mice (Zhang et al., 2010). On the other hand, deletion of Cry1 and Cry2 results in impaired glucocorticoid-receptor-mediated repression of glucocorticoid synthesis (Lamia et al., 2011). This in turn results in increased gluconeogenesis in the Cry1, Cry2 double knockout animals and increased levels of blood glucose in response to both feeding and fasting (Lamia et al., 2011). Deletion of Bmal1 in the liver results in reduced blood glucose levels during the rest period of the daily cycle and increased glucose clearance from the circulation (Lamia et al., 2008). Pancreas-specific deletion of Bmal1 leads to reduced ability of the pancreas to secrete insulin in response to glucose during the active period of the daily cycle (Marcheva et al., 2010). As a result, mice with a dysfunctional pancreatic clock showed impaired glucose tolerance and increased *ad libitum* plasma glucose levels (Marcheva et al., 2010).

The circadian clock has a profound effect on overall energy homeostasis. Exposure of mice to constant light disrupts their rhythms in locomotor activity and leads to obesity without an increase in total food intake (Shi et al., 2013). ClockΔ19 mutant mice on the C57BL/6J background are obese due to hyperphagia and an attenuation of the regular diurnal feeding rhythm (Turek et al., 2005). Mice deficient in Per2 have no glucocorticoid rhythm, lose diurnal feeding rhythm and develop obesity when fed a high fat diet (Yang et al., 2009). Mutation of the core clock gene Per1 that alters the phosphorylation site of PER1 results in a phase advance of food intake by several hours into the rest/sleep period and in obesity (Liu et al., 2014). Further to support the findings in mice with mutations of clock genes, SCN lesions in mice leads to increased body weight and hepatic insulin resistance (Coomans et al., 2013). This suggests that the increased body weight found in mice carrying mutations of clock genes is due to the disruption of the circadian clock and not because of developmental defects. However, the possible developmental effects of mutations/deletions of clock genes have to be formally tested experimentally with the use of post-natal genetic manipulations. A common parameter in all the above animal models of clock disruption that develop obesity is the increase in food intake during the rest/sleep phase, a phase of the daily cycle when mice normally consume little food. Adding further support to the role of food intake timing, disruption of the circadian clock specifically in adipocytes results in obesity also due to attenuation of the normal feeding rhythm (Paschos et al., 2012). Mice with no functional adipocyte clocks eat more than normal during the rest period of the 24 h cycle, without an increase in total daily food intake. Adipocyte clock controls de novo fatty acid synthesis and release to the circulation, which serves as a signal to the hypothalamus to regulate feeding activity (Paschos et al., 2012). Taken together, the studies in clock deficient mice suggest involvement of the circadian clock in the regulation of feeding. Several studies provide support for the role of the time of food intake in body weight homeostasis (Masaki et al., 2004; Fonken et al., 2010; Salgado-Delgado et al., 2010; Hatori et al., 2012; Stucchi et al., 2012; Chaix et al., 2014). Rats forced to eat opposite to their normal eating time develop obesity (Salgado-Delgado et al., 2010). Similarly, a shift of feeding time to the rest phase in a genetic model of irregular feeding behavior (Masaki et al., 2004) or by exposure to light during nighttime increases body weight (Fonken et al., 2010). An increase in the amount of calories consumed during the rest/sleep phase of the daily cycle is causal for the development of obesity during high fat diet feeding (Stucchi et al., 2012; Hatori et al., 2012; Chaix et al., 2014).

Time of day of food consumption appears to be important for energy homeostasis however the mechanisms under which feeding at inappropriate time leads to obesity are not yet understood. Feeding rhythms drive rhythms in liver triglycerides and proteins independent of the circadian clock (Adamovich et al., 2014; Mauvoisin et al., 2014). Feeding at “inappropriate” time entrains those rhythms into a phase opposite to the phase of other physiological rhythms dictated by the master clock. This circadian misalignment may result to inefficiency in energy expenditure and obesity (Mattson et al., 2014). In support of this hypothesis, correction of the feeding time in mice fed a high fat diet rescues the onset of obesity and restores the phase of rhythms in serum metabolites (Chaix et al., 2014). The clinical relevance of the findings in animal studies is highlighted by the increased prevalence of obesity in the human Night Eating Syndrome (Gallant et al., 2012), characterized by a delayed pattern of food intake such that more than 25% of the total daily intake takes place after dinner and into the rest/sleep period (Allison et al., 2010). Some first evidence in humans show that volunteers on a weight loss diet lost 25 percent more weight when they consumed their largest meal earlier in the day (Garaulet et al., 2013). In another study, consuming half of the total daily calories during breakfast as part of a weight loss diet led to greater weight loss compared to high caloric intake during dinner time (Jakubowicz et al., 2013). Further studies are required to elucidate the therapeutic implications of feeding time on energy homeostasis and body weight regulation.

## Conflict of interest statement

The author declares that the research was conducted in the absence of any commercial or financial relationships that could be construed as a potential conflict of interest.
